# Scientific truth: an endangered species

**DOI:** 10.1038/s44319-024-00293-5

**Published:** 2024-10-24

**Authors:** Frank Gannon

**Affiliations:** QIMR Berghofer Medical Research Institute, Brisbane, QLD Australia

**Keywords:** Science Policy & Publishing

## Abstract

Academic research and selection panels need to deal with the problem of predatory journals as these are slowly but inevitably poisoning the scientific record.

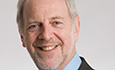

Facts and logic, based on systematic analysis of data, have been the cornerstones of scientific research for centuries. Any hypothesis requires experimental examination, and the results add to our understanding of life. Progress has never been linear—perhaps peristaltic is the more appropriate term—as new information and cross-examination using different experimental systems or more advanced technologies often require a modification of previously held convictions. The essential elements of the system are a combination of anonymous peer review by third-party experts and transparent sharing of the information with the research community for further examination and as a basis for new hypotheses and studies.

Research is a frustrating experience as progress is always slow. Fully confirmed proof of a seductive result can be a difficult challenge and a paper or a preprint published by someone else working on the same topic means that years of work could be scooped at any time. Research careers are constantly in the balance and yet, it is all worthwhile. Science and scientists create knowledge, which is one of the characteristics of being human. They and others transform this knowledge into products and processes that improve society.

But this noble and respected system is under threat by predatory publishers that offer publication in what seems to be bona fide, peer-reviewed journals. Last month alone I had 34 invitations to publish papers on topics that ranged from Surgery to Nursing. And I am not unique—anybody can become a target for such invitations whenever they publish an article. These predatory journals often claim to conduct editorial and peer review but, in fact, merely accept anything submitted to them—for a fee of course. Easy publication without the screen of peer review to demand confirmation, better statistics or correct consideration of alternative interpretations or previously existing data is readily facilitated and, as a result, scientific research is becoming an endangered species as it is slowly but surely being poisoned and distrusted.

The cliché about some bad apples in a barrel applies to the research community as it does to all groups and professions. There are cheats who falsify data to get a tidy result, those who copy without giving credit, those who fill in blanks in experiments because they “know” what the result would be if correctly performed. There are those who misuse statistics, or don’t realise they don’t have the correct settings on instruments. Luckily, there is increased scrutiny of published reports that helps to spot such flawed papers. Pub Peer and Retraction Watch regularly shine a spotlight on questionable publications and help to curate the scientific literature. These internal flaws are nothing novel, and the scientific and publishing process was sufficiently robust to deal with it. The new challenges, however, are external and raise serious questions about science as a reliable repository of truth. The dramatic growth of predatory journals has enabled a “fake science” to stand on the same shelves as real publications. Consider the plight of a scientist who is under pressure to have publications in order to maintain their job or improve their position. An offer of fast acceptance and a reasonable price for publication arrives in their inbox. The journal has an appropriate title and claims to have a reasonable impact factor. A submission would perhaps save a career. But that publication—with results generated under time pressure and lack of appropriate controls—may have a high chance of being preliminary or even incorrect. The predatory journal has no or little editorial control or peer review system. And yet, the paper can be cited with the same credibility as one from a journal with high standards.

Furthermore, some predatory journals have put in place systems to generate citations that benefit the author and the deceptive journal. In the process, it becomes difficult to distinguish phoney from real science. As the contamination grows and intertwines with quality-controlled research, all science loses its value. Retraction Watch states that 10,000 articles were retracted in 2023 and that hundreds of journals were exposed as being established to dupe authors to pay for an easy passage to print without a proper peer review system. Like the many-headed hydra, each one cut down is replaced and an offer from a new journal appears in the inbox.

If science was a place for genial amateurs as in the Victorian days, none of this would have a big impact on society. But today, scientific information is the basis of policy decisions and the development of new treatments. When some published research papers are not robust, it is easy for those with preconceived ideas to find one that will support their claims about the environment, climate, the causes of disease, the evils of vaccines or the best way to manage a pandemic. “The science has said…” became a Covid-era refrain. But if one can be selective about which scientific “result” to use and if the options include phoney science, then science itself is no longer reliable as a guide. As the amplification of untruths expands in civil society, it becomes urgent to recognise the scale of the problem and the disastrous consequences that follow from a society that is based on opinion and not on fact/science. We are close to the point where the endangered species is threatened by extinction.

As the core problem is the difficulty of distinguishing robust research from flimsy papers, it would seem that it is time to turn the tide. The primary motivation for those who cut corners and submit to journals without established due process of peer review, is to advance their careers. This suggests that the best way to start to reverse the trend is for research granting bodies and appointment boards to exclude from their consideration papers from journals that do not meet some defined criteria. By way of example, the criteria for inclusion in the databases of Clarivate or PubMed entail “Scientific Quality, Content Relevance: Publication Ethics, Regular Publication, Editorial Board, Peer Review Process, Indexing Standards, Accessibility, Citation and Impact”. Applicants could, for instance, have to certify that their papers appeared in journals that were accepted in those databases. The alternative is that journals would seek certification by an organisation that makes sure that publishers meet these criteria; a selection committee or funding agency could then require such a tag of compliance. In time, the cancerous growth of concocted papers could be removed from the information system, and those who have built reputations based on such fiction should be exposed. If we do not take steps now, science will become increasingly useless, and the world will suffer the consequences.

## Supplementary information

Peer Review File

