# Supplementary material for: Scientific truth: an endangered species
Source: EMBO Rep. 2024 Oct 24;25(12):5225–6. doi: 10.1038/s44319-024-00293-5 (PMC11624274; doi:10.1038/s44319-024-00293-5)
Supplement: Supplementary file 1 — Peer Review File [file 44319_2024_293_MOESM1_ESM.pdf]

EMBO reports

Scientific Truth: An endangered species

Frank Gannon

Corresponding author(s): Frank Gannon (FGannon@qimrberghofer.edu.au)

Review Timeline:

Submission Date:	14th Oct 24
Accepted:	14th Oct 24

Editor: Holger Breithaupt

Transaction Report: This article type is not peer reviewed.
